# Neoadjuvant chemotherapy for primary adenocarcinomas of the urinary bladder: a single-site experience

**DOI:** 10.1186/1471-2490-15-3

**Published:** 2015-01-28

**Authors:** Bin Yu, Jin Zhou, Hongzhou Cai, Ting Xu, Zicheng Xu, Qing Zou, Min Gu

**Affiliations:** Department of Urologic Surgery, Affiliated Cancer Hospital of Jiangsu Province of Nanjing Medical University, Nanjing, China; Department of Urology, First Affiliated Hospital of Nanjing Medical University, Nanjing, China; Department of Hospital Infection Control, Affiliated Cancer Hospital of Jiangsu Province of Nanjing Medical University, Nanjing, China

**Keywords:** Neoadjuvant chemotherapy, Primary adenocarcinomas of bladder, Survival

## Abstract

**Background:**

Adenocarcinoma of the urinary bladder is a rare malignancy. Radical surgery is suggested as the best available treatment for early-stage disease, but there is currently no consensus on standard chemotherapy regimen for advanced stage. We assessed the feasibility and effect of neoadjuvant chemotherapy with gemcitabine and cisplatin (GC) plus S-1 for patients with locally advanced primary adenocarcinomas of the urinary bladder.

**Methods:**

Six patients with locally advanced urachal or non-urachal (n = 3, each) primary adenocarcinoma of the bladder were treated from October 2010 to October 2013 at a single center. All the patients were treated with 3 cycles (21d, each) of GC plus S-1 (gemcitabine, 1000 mg/m^2^, days 1 and 8; cisplatin, 70 mg/m^2^, day 2; and S-1, 50 mg bid, day 1-14). After neoadjuvant chemotherapy, patients with urachal cancer were treated with *en bloc* radical cystectomy and umbilectomy; the remaining 3 patients were treated with cystectomy.

**Results:**

All patients successfully completed the neoadjuvant chemotherapy without serious side effects. Two patients were assessed as complete response, 2 as partial response, 1 as stable disease and 1 as progressive disease.

**Conclusions:**

Despite the limitations of a small study population, the GC plus S-1 regimen for locally advanced primary adenocarcinoma of the urinary bladder was effective, and facilitated the success of surgery to a certain extent. Short follow-up time was also a limitation of our study. More studies are needed to evaluate the results.

## Background

Primary adenocarcinomas of the bladder account for less than 2% of primary bladder cancers, and are rarely encountered [1, 2]. Primary adenocarcinomas of the urinary bladder can be subdivided into urachal and non-urachal adenocarcinoma. Radical surgery is considered the best available treatment, but the 5-year survival rates (11%-61%) have not been satisfactory [3]. Patients with urachal adenocarcinoma often present with higher stage disease because the disease arises out-side of the bladder and causes fewer symptoms before its invasion of surrounding organs. For patients with locally advanced primary adenocarcinomas of the bladder, neoadjuvant chemotherapy can be a good option: it helps downstage the cancer before radical surgery, eradicates potential micrometastases, and may avoid local and distant failure [4, 5]. However, primary adenocarcinomas of the urinary bladder are so rare that experience of chemotherapy is limited [6]. The National Comprehensive Cancer Network (NCCN; version 2.2014) suggests that chemotherapy with methotrexate, vinblastine, doxorubicin, and cisplatin (MVAC) is not effective for primary adenocarcinomas of the urinary bladder, and according to some reports a 5-fluorouracil (5-FU)-based regimen should be tried [6, 7]. Furthermore, gemcitabine plus cisplatin (GC) has proved viable as first-line chemotherapy for urothelial (transitional) cell carcinoma with overall survival comparable to that of MVAC but with fewer side effects [8]. Herein, we report our experience in treating 6 cases of locally advanced primary adenocarcinomas of the bladder, 3 urachal and 3 non-urachal.

## Methods

This prospective observational study was approved by the Institutional Review Board of Nanjing Medical University, Nanjing, China. Each patient provided written informed consent before treatment. Six patients with pathologically diagnosed locally advanced primary adenocarcinoma of the bladder were treated between October 2010 and October 2013 at Affiliated Cancer Hospital of Jiangsu Province of Nanjing Medical University. The patients’ demographics and clinical data were collected. All the pathology gained through biopsy via cystoscopy was confirmed as adenocarcinoma before chemotherapy. Computed tomography of cranial, chest and abdomen was done to exclude the metastatic adenocarcinoma. All patients were confirmed primary adenocarcinoma of the bladder at a locally advanced stage, with clinical stage ≥ T_2_, without distant metastasis, and with or without lymph node metastasis. All the patients were treated with GC plus S-1 chemotherapy for 3 cycles, 21 d/cycle: gemcitabine, 1000 mg/m^2^, days 1 and 8; cisplatin, 70 mg/m^2^, day 2; and S-1, 50 mg bid, days 1-14. The adenocarcinomas were classified as as urachal or non-urachal prior to surgery. Tumors were considered urachal if the tumor was located at the dome of the bladder rather than the anterior bladder wall, with the most critical features being the presence of a sharp demarcation between the tumor and the surface epithelium, and the exclusion of a primary adenocarcinoma elsewhere. Patients with urachal bladder cancer were treated with *en bloc* radical cystectomy and umbilectomy. Non-urachal patients were treated with cystectomy after neoadjuvant chemotherapy. The response of the target lesions to treatment was evaluated by computed tomography scan, in accordance with the Response Evaluation Criteria In Solid Tumors (RECIST 1.1) guidelines: complete response (disappearance of target lesion); partial response (≥30% decrease in the sum of the longest diameter of the target lesion, from baseline); stable disease (between a partial response and progressive disease); and progressive disease (≥20% increase in the sum of the longest diameter of the target lesion, from nadir) [9]. The patients were followed, beginning at diagnosis.

## Results

Six patients were enrolled in this study: 5 men and 1 woman, aged 49 to 79 years (mean 62.5 y; Table 1). Hematuria and lower abdominal pain were the most frequently reported symptoms at presentation. Specifically, 4 patients presented with hematuria and 3 with lower abdominal pain (1 patient with both symptoms). One, 3, and 2 patients were in stages T_2_, T_3_, and T_4_, respectively. One month after the 3 cycles of neoadjuvant chemotherapy, 2, 2, 1, and 1 patient were evaluated as complete response, partial remission, stable disease, and progressive disease, respectively. No patient stopped chemotherapy because of side effects. Three patients were judged to have non-urachal adenocarcinoma. Two of these received *en bloc* radical cystectomy and umbilectomy. One patient refused surgery. The 3 patients with non-urachal adenocarcinoma were treated by cystectomy. After surgery, pathology confirmed pure primary adenocarcinoma in 5 patients.

**Table 1:** **Case summaries of 6 patients with primary adenocarcinomas of the urinary bladder**

Patient	Age	Gender	Symptom	Tumor	Stage	Response ^a^	PFS, mo ^b^	Survival, mo
1	71	Male	Pain	Non-urachal	T3b	Complete	3	7
2	68	Male	Hematuria	Non-urachal	T2b	Partial	>18	18^c^
3	79	Female	Hematuria	Non-urachal	T4b	Stable disease	6	12
4	67	Male	Pain	Urachal	T3b	Complete	>20	20^c^
5	51	Male	Hematuria	Urachal	T3b	Partial	>10	10^c^
6	49	Male	Pain and hematuria	Urachal	T4a	Progressive disease	>6	6^c^

^a^After neoadjuvant chemotherapy; ^b^progression-free survival; ^c^still alive.

Four patients experienced grade 2 myelosuppression. Three patients had transient elevated serum creatinine (averaging 1.5-fold normal). Three patients had nausea, vomiting, and loss of appetite. All the patients recovered from the side effects after completion of chemotherapy.

## Discussion

Adenocarcinoma of the urinary bladder is a rare malignancy with a poor prognosis. Patient survival is similar to that of muscle-invasive urothelial carcinoma [10]. Unlike other cancers, there is currently no consensus regarding a standard chemotherapy regime for adenocarcinoma of the urinary bladder. In the present study, we used GC plus S-1 for neoadjuvant chemotherapy. Johnson et al. [11] reported that adenocarcinomas of the urinary bladder have a clinical behavior similar to that of urothelial (transitional) cell carcinoma of the bladder, and GC as first-line chemotherapy for urothelial cell carcinoma has fewer side effects and similar overall survival compared with MVAC. 5-FU is widely used in the treatment of adenocarcinomas (such as stomach cancer), and Logothetis et al. [6] reported using 5-FU to treat urachal cancers, but continuous 5-FU infusion has recently been replaced by S-1, to avoid some of the inconveniences and adverse effects of 5-FU [12, 13]. S-1 is a novel oral Xuoropyrimidine derivative that consists of tegafur, 5-chloro-2, 4-dihydropyrimidine (CDHP), and potassium oxonate.

All the 6 patients of the present study received GC plus S-1 for 3 cycles, and no patient failed to complete the cycles because of side effects. The most severe side effect was grade 2 myelosuppression such as leucopenia and thrombocytopenia. This was resolved within 2 to7 days after symptomatic treatment with granulopoietin. More severe side effects can be treated by blood transfusion. No patient suffered renal function impairment because of chemotherapy. Three patients experienced transient elevated serum creatinine (averaging 1.5-fold normal). However, this also returned to normal after the end of treatment. Thus, the safety of this chemotherapy regime was acceptable.

In the present study, we applied GC plus S-1. Two patients had a partial response, and 2, 1, and 1 patient had complete response, stable disease, and progressive disease, respectively. In contrast, a study conducted by the MD Anderson Cancer Center (MDACC) used combinations of 5-FU and cisplatin and reported a 33% response rate [7]. Thus, our results are encouraging. Several factors may contribute to the difference in response rate. Firstly, the composition of patients is different. The patients in MDACC report are pure with urachal cancers, while in our study, NU adenocarcinoma patients are included. Secondly, the differences in the predominant ethnicities of the study populations (Houston, TX cf. Nanjing, China) may have influenced the response rate to chemotherapy. Many studies report that genetic polymorphisms exist in different races, especially in drug-metabolizing enzymes, drug targets, and drug receptors [14, 15]. These genetic polymorphisms may have contribution to the response rate. Compared with western populations, the patients in China have more attention from their family. Better food and family care may encourage the nutrients and psychological healthy of patients. More importantly, adenocarcinomas of the urinary bladder are rare and all studies are limited in the small cases. The choice of chemotherapy regimens has been based largely on case reports and single institution experiences and the statistical power is limited.

After 3 cycles of neoadjuvant chemotherapy, 5 patients received surgery. The surgeries were straightforward and uneventful. Considering neoadjuvant chemotherapy may lead to increased edema and adhesion of the bladder to the surrounding tissue, increasing the difficulty of the operation. However, no unusual bleeding or postoperative infection was observed in our cases. Patient 1 rejected surgery because he had no medical insurance and could not bear any further financial burden. This patient had complete response after 3 cycles of chemotherapy, but after 3 months, the tumor recurred.

The majority of relevant studies have indicated that tumor stage is a highly significant predictor of outcome [16, 17]. The shorter follow-up time, small case number, and the heterogeneity of patient 1 (who refused surgery and even other treatments) handicapped our analysis in this study. However, our results are enough to warrant further clinical studies with a larger number of patients with primary adenocarcinomas of the urinary bladder, to evaluate the efficacy of GC plus S-1 combination chemotherapy for neoadjuvant chemotherapy, and even for adjuvant chemotherapy.

Despite the limitations, the preliminary results of this study show that chemotherapy with GC plus S-1 for primary adenocarcinomas of the urinary bladder is effective. For patients at a locally advanced stage, this regimen effectively downstaged the tumor and may eradicate potential micrometastases, without increasing the difficulty of surgery.

## Conclusions

Chemotherapy with the GC plus S-1 regimen for patients with primary adenocarcinomas of the urinary bladder is effective. To a certain extent, neoadjuvant chemotherapy facilitates surgery of patients at the locally advanced stage. Our treatments should be investigated in a randomized controlled trial to confirm these outcomes.
